# A Novel mHealth App for Smokers Living With HIV Who Are Ambivalent About Quitting Smoking: Formative Research and Randomized Feasibility Study

**DOI:** 10.2196/58063

**Published:** 2024-07-08

**Authors:** Jennifer B McClure, Jaimee L Heffner, Chloe Krakauer, Sophia Mun, Sheryl L Catz

**Affiliations:** 1 Kaiser Permanente Washington Health Research Institute Seattle, WA United States; 2 Kaiser Permanente Bernard J Tyson School of Medicine Pasadena, CA United States; 3 Fred Hutchinson Cancer Center Seattle, WA United States; 4 Betty Irene Moore School of Nursing University of California, Davis Sacramento, CA United States

**Keywords:** HIV, tobacco, nicotine, smoking cessation, mobile health, mHealth, motivation, ambivalence, app, mobile phone

## Abstract

**Background:**

More people who smoke and are living with HIV now die from tobacco-related diseases than HIV itself. Most people are ambivalent about quitting smoking and want to quit someday but not yet. Scalable, effective interventions are needed to motivate and support smoking cessation among people ambivalent about quitting smoking (PAQS) who are living with HIV.

**Objective:**

This study aims to develop an app-based intervention for PAQS who are living with HIV and assess its feasibility, acceptability, and potential impact. Results of this study will inform plans for future research and development.

**Methods:**

In phase 1, PAQS living with HIV (n=8) participated in user-centered design interviews to inform the final intervention app design and recruitment plan for a subsequent randomized pilot study. In phase 2, PAQS living with HIV were randomized to either a standard care control app or a similar experimental app with additional content tailored for PAQS and those with HIV. Participants were followed for 3 months. Feasibility focused on recruitment, retention, and participants’ willingness to install the app. The study was not powered for statistical significance. Indices of acceptability (satisfaction and use) and impact (smoking behavior change and treatment uptake) were assessed via automated data and self-report among those who installed and used the app (n=19).

**Results:**

Recruitment for both study phases was a challenge, particularly via web-based and social media platforms. Enrollment success was greater among people living with HIV recruited from a health care provider and research registry. Once enrolled, retention for the phase 2 randomized study was good; 74% (14/19) of the participants completed the 3-month follow-up. Phase 1 findings suggested that PAQS living with HIV were receptive to using an app-based intervention to help them decide whether, when, and how to stop smoking, despite not being ready to quit smoking. Phase 2 findings further supported this conclusion based on feedback from people who agreed to use an app, but group differences were observed. Indices of acceptability favored the experimental arm, including a descriptively higher mean number of sessions and utilization badges. Similarly, indices of potential impact were descriptively higher in the experimental arm (proportion reducing smoking, making a quit attempt, or calling free tobacco quitline). No participants in either arm quit smoking at the 3-month follow-up.

**Conclusions:**

On the basis of this formative work, PAQS living with HIV may be receptive to using a mobile health–based app intervention to help them decide whether, when, or how to stop using tobacco. Indices of acceptability and impact indicate that additional research and development are warranted.

**Trial Registration:**

ClinicalTrials.gov NCT05339659; https://clinicaltrials.gov/study/NCT05339659

## Introduction

### Background

More than 1 million Americans [1] and 39 million people globally live with a diagnosis of HIV [2]. With the widespread use of antiretroviral therapy (ART), life expectancy for these individuals has substantially improved. However, people who smoke and are living with HIV have a 2-fold greater mortality compared to people living with HIV who do not smoke [3-13]. In fact, smokers living with HIV now lose more life years to tobacco-related diseases than to their HIV infection [14]. This is due in part to increased risk for AIDS-defining illnesses such as bacterial pneumonia, lung cancer, and other malignancies [3-13] and to potentially poorer viral and immunologic responses to ART, all of which result in a higher risk of developing AIDS [3,15-17]. Quitting smoking can reduce the incidence of morbidity and mortality among people who smoke and are living with HIV and improve their quality of life [18]. As such, promoting smoking cessation among people living with HIV is an important public health goal.

The US treatment guidelines recommend people who smoke and are living with HIV be offered evidence-based treatment following best practices for treating nicotine dependence, which combines advice to quit, skills-based training (via counseling or self-help materials), and pharmacotherapy (eg, nicotine replacement therapy [NRT], varenicline, and bupropion) [18,19]. Despite this recommendation, based on several systematic reviews of the literature, it remains unclear whether standard care treatment is efficacious among smokers living with HIV [20-22], prompting the US National Cancer Institute to call for research to develop and evaluate evidence-based smoking cessation interventions for people living with HIV [23]. This study was funded in response to that call and seeks to not only develop a novel intervention for smoking cessation targeted to people living with HIV but also to create an app-based intervention for those who are ambivalent about quitting. People ambivalent about quitting smoking (PAQS) want to quit someday but not yet. Most people who smoke (approximately 70%) meet this criterion [24]. Research also shows high rates of ambivalence about quitting smoking among people living with HIV [25].

We are not aware of any interventions designed specifically for PAQS who have HIV, but there is a good reason to expect that this may be a viable intervention strategy. For example, studies with PAQS recruited from the general population demonstrate that PAQS are open to receiving support and enrolling in smoking-focused intervention trials [26-30], despite their ambivalence about quitting smoking in the near term. In addition, a recent meta-analysis of 22 studies found that interventions designed for PAQS can be as effective as those targeted to people who are ready to quit [29].

Furthermore, we recently found that PAQS were receptive to using an app-based intervention to help them decide whether, when, and how to stop smoking. Relative to a control app that included access to standard care advice and access to treatment for people ready to quit, the experimental app (called GEMS) had higher engagement (mean sessions 19.9, SD 16.2 vs 7.3, SD 6.6), was associated with greater requests for either NRT or quitline counseling accessed through the app (36% vs 10%), and had a higher 7-day point prevalence abstinence at the 3-month follow-up (14.7% vs 6.9%) in a randomized pilot study [31]. Recent studies have also found app-based smoking interventions to be feasible and acceptable to people living with HIV who were ready to quit [32,33]. Taken together, these recent studies suggest that it is reasonable to explore the use of an app-based intervention to change smoking behavior among PAQS living with HIV. If this intervention is acceptable and feasible, it could potentially improve the reach of evidence-based smoking cessation services for this population of smokers.

### Objectives

The ultimate aim of this research is to develop an effective, evidence-based, app-delivered smoking cessation intervention for PAQS living with HIV. The goals of this formative work were to inform and refine the existing GEMS intervention content for use with PAQS living with HIV (now called GEMS+), understand the potential acceptability of the intervention, explore the feasibility of conducting a future large-scale randomized trial, and collect preliminary data on the potential impact of the intervention. Findings from this work have implications for future enhancements to the GEMS+ app, development of other app-based interventions targeting both PAQS and smokers living with HIV, and the feasibility of a future large-scale effectiveness trial.

## Methods

### Setting

Research activities were conducted at the Kaiser Permanente (KP) Washington Health Research Institute. The study is registered with ClinicalTrials.gov (NCT05339659).

### Ethical Considerations

This study was approved by the KP Washington Institutional Review Board (reference #2021).

### 2-Phase Study Design and Human Participants’ Considerations

This work was conducted in 2 sequential phases. During phase 1, we conducted user-centered interviews with PAQS living with HIV to iteratively inform key decisions regarding the study recruitment materials, recruitment methods, and the app content and design. Interviews were conducted between September and December 2021. Study materials were adapted from those previously developed for and tested with PAQS [31,34].

During phase 2, we conducted a randomized, parallel, 2-arm proof of concept pilot study to assess the feasibility of recruiting study participants, the acceptability of the app-based smoking intervention based on automated use data and self-report, and the impact of the experimental intervention use on key indicators of treatment efficacy at 1 and 3 months after randomization. Study data were collected between May 2022 and May 2023. All phase 1 and phase 2 research participants provided informed consent to participate. Because study data were identifiable and not anonymous, multiple strategies were used to protect participants’ confidentiality, including storing survey data behind a secure firewall, tracking participants with IDs and not names, and not saving identifiable information within the app or on participants’ phones. No generative artificial intelligence was used to conduct this research or prepare this report of findings.

### Phase 1: User-Centered Design Interview Screening and Methods

Participants were recruited through Facebook (Meta Platforms, Inc) advertisements, through ResearchMatch, and from KP Washington members. Individuals were screened by phone and were eligible if they self-reported an HIV diagnosis, were aged ≥18 years, could read and speak in English, smoked at least 100 lifetime cigarettes, smoked in the past week, wanted to quit smoking someday but not in the next month, owned a smartphone and used apps on their phone at least once a week, had a computer with a camera and internet connection, and had a US mailing address. Recruitment continued until a demographically diverse cohort screened eligible, consented, and enrolled; however, the final sample was also influenced by the limited study timeline and available resources.

Interviews were conducted remotely using a secure, Health Insurance Portability and Accountability Act–compliant video software. Participants were shown sample study recruitment materials (advertisement text and graphic imagery), screenshots from the original GEMS app, and wireframe mock-ups for new content and images planned for the new app based on expert input. The latter included images of a diverse group of models, content designed for people living with HIV (ie, risks of smoking and benefits of quitting for people living with HIV), and information addressing questions raised by participants in our prior research with smokers living with HIV [35-37]. Participants also reviewed several options of names for the new app. Session recordings were transcribed and reviewed for key themes, which informed iterative refinement of the phase 2 study recruitment and intervention materials. Participants received a US $50 gift card as a token of appreciation for completing the interview.

### Phase 2

#### Randomized Pilot Study Recruitment and Screening

##### Recruitment

Pilot study participants were recruited via social media advertisements, direct outreach to KP Washington members living with HIV and people enrolled in ResearchMatch, partnership with various HIV or AIDS service organizations, and flyers placed in community health clinics in the Seattle area. Those interested in learning more about the study were invited to contact the study staff to be screened for eligibility by phone.

##### Eligibility and Fraud Detection Criteria

Individuals were eligible for the pilot study if they were aged ≥18 years, were comfortable reading and speaking in English, smoked at least 100 lifetime cigarettes, smoked at least 5 cigarettes a day and smoked in the past week, were interested in quitting smoking someday but not in the next month, owned an Android or Apple smartphone and used it at least weekly, were willing to download and install the app, and self-reported an HIV diagnosis. To deter fraudulent participation, participants were required to have a verifiable US-based mailing address and US phone number that was not a Google Voice number. Individuals were excluded if they reported using a virtual private network on their phone; had a lifetime history of dementia, bipolar disorder, or schizophrenia; had contraindications for NRT use; had visual impairments that precluded their ability to read content on their phone and had no adaptive devices; if another member of their household was already enrolled in the study; or if they participated in the phase 1 design interviews. KP members were prescreened via electronic health records before invitation to confirm HIV diagnosis and to exclude people with medical exclusions noted above*.*

#### Randomization and Pilot Study Flow

After completing the baseline survey, participants were randomly assigned to 1 of the 2 app versions (control or experimental) using an automated, block-stratified randomization scheme to ensure balanced representation of lighter versus heavier smokers (≥15 cigarettes per day vs ≤14 cigarettes per day). Anyone who failed to install the app after 3 days was offered technical assistance by phone.

Once the app was installed, participants entered a unique code that unlocked their assigned intervention (control vs experimental) and allowed use tracking. Participants then viewed a welcome screen and a brief tutorial. Intervention content could be accessed ad-lib until completion of the 3-month follow-up survey, after which access was remotely deactivated.

#### Pilot Study Intervention Content and Functionality

##### Overview

Both versions of the GEMS+ app (control and experimental) had a similar design and largely identical content, except the experimental version also included content designed for PAQS living with HIV. As a result, both groups received an active smoking cessation intervention. The rigorous design allows participants to be blinded to their arm assignment and for observed differences between arms to be attributed to the additional experimental content.

##### Control App Content and Features Common to Both App Versions

The control app content was based on standard, evidence-based treatment grounded in the US Public Health Service Guidelines for Treatment of Nicotine Dependence [19] and cognitive behavioral therapy for smoking cessation [38], with additional content and features informed by user-centered design work previously conducted by our team [39-41] and our phase 1 results. Control messaging briefly acknowledged that users were not ready to stop smoking but otherwise focused on how to stop smoking when ready.

The content and functionality of the control app was identical to that recently developed and pilot-tested with PAQS in the prior GEMS study [31]. It featured a Quit Guide, including advice to quit smoking, didactic information (eg, what is nicotine withdrawal and how does pharmacotherapy work), and a 6-step guide on how to quit (eg, how to choose a stop-smoking medicine, how to set a quit date, how to prepare for your quit date, what to do on your quit date, and how to stay the course and prevent relapse). Participants who were ready to stop smoking could call a tobacco quitline and enroll in free standard care services available to US residents. Other content included a daily cigarette tracker, a calculator for estimating how much money could be saved by quitting smoking, and 2 sets of peer advice presented through short testimonials: 1 set offering motivational encouragement for quitting smoking and 1 set modeling how to counter common excuses for smoking or not quitting. Participants could also use an in-app journal feature to take notes.

Control participants could earn up to 10 badge rewards based on use. After 6 badges were earned, participants could request a free 2-week trial of NRT to help them stop smoking.

##### Experimental App Content and Unique Features

The experimental app mirrored the control app’s design, content, and functionality with 5 exceptions. First, the home page of the experimental version highlighted a series of 10 cognitive and behavioral exercises called personal experiments (Textbox 1). Each of these exercises was designed to help users clarify their values, build and strengthen their motivation for reducing or quitting smoking, or enhance their self-efficacy for changing smoking behavior by learning specific skills that could help them manage cravings and resist the urge to smoke. All content was designed for PAQS, with some content specifically tailored to PAQS living with HIV. Participants also received SMS text message reminder prompts to initiate or complete each experiment. Second, the Quit Guide was moved to the Toolbox, making it less prominent than in the control arm, but still accessible from the home page navigation bar. Third, the peer advice and testimonials addressed similar topics as in the control arm, but some narrative content was tailored for people living with HIV (eg, addressed issues related to ART and smoking or the impact of smoking on immune functioning and AIDS). Fourth, the Toolbox included an additional section that contained factual information about smoking and HIV (“Just the Facts”). Fifth, participants in the experimental arm could earn up to 21 total badges: 10 for completing each of the personal experiments, 1 for viewing the HIV information in the Toolbox, and 10 for viewing the other program features common with the control app (eg, Quit Guide, cigarette tracker, and peer testimonials). As in the control arm, after 6 badges were earned, users could request a free trial of NRT.

The theoretical rationale for the personal experiments, an overview of their design and flow, and preliminary formative research testing their acceptability and potential impact with PAQS have been previously reported [31,34]. Briefly, the experiments and overall intervention concept are grounded in empirically validated recommendations for treating nicotine dependence [19] and several complementary motivation and behavior change theories (eg, the PRIME theory of motivation [42,43], cognitive behavioral therapy, acceptance and commitment therapy, and social cognitive theory [44-46]). The personal experiments were also informed by the model of persuasive design proposed by Fogg [47], which suggests that when people have low motivation for change (as with PAQS), the behaviors they are expected to engage in should be simple (ie, require low ability) and coupled with extrinsic triggers to prompt engagement (ie, reminder prompts). The specific behavioral goals and skills targeted in each personal experiment are summarized in Textbox 1.

Experiments 1 and 2 could be completed in the moment. Experiments 3 through 10 were intended to be practiced over a 24-hour period. After each experimental exercise, participants completed a brief series of reflective questions. Completion of each experiment unlocked the next, thereby allowing users to progressively build on the skills already learned to prepare for their practice quit attempt (the last experiment). However, if an experiment was not completed after a specified period, the next automatically unlocked. Emphasis was placed on trying each experiment and observing what one learned, as opposed to skill mastery, to reduce perceptions of failure, which could undermine continued participation.

**Goals and skills for personal experiments within the GEMS+ app.**

**Targeted skills and goals for each experiment**

- Your 75th Birthday
  Clarify one’s values and health goals.
- Whatcha Know?
  Test one’s knowledge about the effects of smoking on HIV.
- Explore Your Motivation
  Identify personal reasons for quitting.
  Build motivation for change.
- Track Your Triggers
  Identify high-risk situations for smoking.
  Inform future problem-solving and preparation for quitting.
- Box Breathing
  Learn deep breathing as a tool for stress reduction and craving management.
  Build self-efficacy for managing cravings and motivation for quitting.
- Mastering the Unlit Cig
  Mindful acceptance. Learn to let urges pass without smoking.
  Enhance self-efficacy for managing cravings and motivation for quitting.
  Create positive outcome expectations.
- Make Smoking Boring
  Stimulus control. Learn to reduce the reinforcing effects of smoking.
  Enhance self-efficacy and motivation for quitting.
- It’s Your Choice
  Cognitive restructuring. Think of not smoking as a positive choice rather than a deprivation.
  Support self-efficacy and create positive outcome expectations for quitting.
- Cut it Out
  Successive approximation. Reduce daily smoking.
  Support self-efficacy and positive outcome expectations.
- Put it Into Practice
  Put all skills into practice with a 24-hour “practice” quit.
  Enhance self-efficacy, motivation, and positive outcome expectations.

#### Pilot Study Key Outcomes and Baseline Measures

##### Sources

Participants completed self-report surveys at baseline, 1 month, and 3 months after enrollment and received a US $25 gift card for each survey completed. Surveys were conducted over the internet using REDCap (Research Electronic Data Capture; Vanderbilt University). Persons who did not complete the person in a timely way were contacted by phone to be interviewed. App use was assessed with automated, time-stamped data. Experimental participants’ ratings of each personal experiment’s helpfulness was also collected in real time following completion of each exercise.

##### Baseline Assessment

*Baseline measures* included participant demographics; use of other smoking cessation apps; use of other forms of tobacco and e-cigarette; and self-reported lifetime diagnosis or treatment for depression, anxiety, bipolar disorder, schizophrenia, alcohol use disorder, or drug use (assessed as a single yes or no for any of the listed conditions). Nicotine dependence was assessed with the Fagerström Test of Nicotine Dependence [48]. Problem drinking was assessed with the Alcohol Use Disorders Identification Test-Consumption [49]. Frequency of marijuana or cannabis use in the past year was assessed with a single item. Response options were never, less than monthly, monthly, weekly, and daily or almost daily.

##### Outcomes of Interest

We examined a range of pilot study outcomes to inform the feasibility, acceptability, and potential impact of the experimental app relative to the control version. Key feasibility outcomes included our ability to recruit and retain PAQS-HIV and participants’ willingness to install and use the app. Measures of acceptability included satisfaction ratings and use metrics assessed as total number of user sessions, duration of app use calculated as number of days between installation and last use, number of reward badges earned, number of participants who earned enough badges to request free NRT, the number of participants who used each app feature common to both versions, and the number of participants who completed each personal experiment in the experimental arm. Satisfaction with participants’ assigned app and advice was assessed using a 5-point Likert scale ranging from 1=“not at all” to 5=“extremely” and by asking if they would refer the app to a friend. In the experimental arm, users also rated the helpfulness of each personal experiment using a 5-point Likert scale ranging from 1=“not helpful” to 5=“very helpful.” The latter ratings were made in real time following completion of each personal experiment.

Key behavioral outcomes were the proportion of participants who reported a ≥50% reduction in smoking from baseline to the 3-month follow-up, presence of a self-reported quit attempt lasting at least 24 hours, self-report of no smoking during the past 7 days (7-day point prevalence abstinence), the proportion of participants who clicked on the Call Now button to access free quitline counseling, and requests for free NRT among those who earned it.

Additional outcomes of interest that could serve as potential cognitive intermediaries of future behavior change included motivation and self-efficacy, each assessed for smoking fewer cigarettes a day and for quitting smoking among those continuing to smoke at the 1-month follow-up using with a 10-point Likert scale ranging from 1=“not at all” to 10=“extremely.”

#### Sample Size

It is recommended that pilot trials of behavioral treatment interventions include approximately 15 to 30 participants per arm to test feasibility [50]. As such, the original target sample for the randomized pilot was set at 25 per arm or 50 total participants.

#### Pilot Study Data Issues

There were no app software updates during the study.

### Phase 1 and 2: Data Analyses

#### Analytic Samples and Missing Data

All enrolled phase 2 pilot participants (n=23) were used to evaluate the study recruitment methods. App installation was assessed among those who were consented, enrolled, and not subsequently deemed ineligible due to the fraud detection protocol (22/23, 96%). Additional phase 2 analyses used an *a priori* defined modified intent-to-treat approach and were limited to randomized participants who installed the app and who were not excluded per the fraud protocol (19/23, 83%). Everyone in this phase 2 analytic sample contributed automated app use data, but self-report data were subject to missingness. One experimental arm participant used the app behind a virtual private network. As a result, time-stamped event data captured through the commercial tracking platform, Countly, were not available, but installation data, number of reward badges, number of sessions, experiments completed and postexperiment survey data, data for use of the cigarette tracker, and duration of app use were available through the study’s custom backend database.

Comparisons of app satisfaction ratings were restricted to participants who earned at least 1 utilization badge by the time of the survey. Per convention, missing smoking outcomes were conservatively imputed as smoking [51]. For consistency, we used a similar approach when analyzing 24-hour quit attempts (ie, missing data were imputed as not making a quit attempt). For all other outcomes, analyses used complete cases without imputation.

#### Analyses

Descriptive statistics were used to characterize the phase 1 and phase 2 samples and key outcomes of interest. For continuous outcomes, we fit linear regression models to estimate expected differences in outcomes between randomization groups. For precision, we adjusted for the number of cigarettes smoked per day at baseline and, when applicable, baseline values of the outcome. We fit separate regression models for outcomes collected at each of 1- and 3-month postenrollment surveys.

Due to small sample sizes and rarity of many binary outcomes, risk differences (RDs) were estimated using observed arithmetic differences in rates between randomization groups. Exact CIs were calculated following the inductive method proposed by Wang [52]; therefore, no large sample statistics were required. Analyses were conducted in R (version 4.0.2; R Foundation for Statistical Computing) [53]. The study was not powered for statistical significance; therefore, all results are considered descriptive and not definitive.

## Results

### Phase 1: User-Centered Design Interviews

#### Participants

Interviewees were predominantly male (6/8, 75%). Of the 8 participants, 4 (50%) identified as non-Hispanic White, 2 (25%) as Black, and 2 (25%) as Hispanic or Latino. Moreover, 63% (5/8) of the participants identified as gay or lesbian, 25% (2/8) as bisexual, and 13% (1/8) as pansexual. The median age of the participants was 50 (range 28-68) years. Participants reported smoking for 10 to >40 years, and number of cigarettes smoked ranged between half a pack and one and a half packs of cigarettes per day. All participants (8/8, 100%) reported using social media such as Facebook, Instagram (Meta Platforms, Inc), and Twitter (subsequently rebranded as X; X Corp), and several participants (5/8, 63%) routinely used HIV-specific social media forums.

#### Key Feedback

Participants were invited to provide feedback to inform the recruitment plan for the phase 2 randomized pilot, the app name, and final app intervention content. Most participants (6/8, 75%) said they would click on a social media advertisement seeking smokers living with HIV to help test a new app. All but 1 (7/8, 87%) participant felt identifying KP in the advertisement would lend credibility and make them more likely to click on the recruitment link. One person felt mentioning KP could be a deterrent because people might assume they needed to be a KP member to participate. As a result, the final advertisement content identified KP as the organization conducting this work but included a statement that people did not need to be a KP member to enroll.

Given the stigma associated with HIV, we also sought guidance about how overt or subtle the app and recruitment advertisements should be about HIV. Participants advised that messaging in and about the app should make it clear that the app is for people living with HIV (eg, “Be explicit. Don’t hide us.” [participant 44] and “It’s almost offensive to not just be to the point, as most people with HIV don’t have shame around it these days.” [participant 15]). Final advertisement text was adjusted accordingly.

Next, we assessed participants’ reaction to the general concept for the intervention. All participants (8/8, 100%) liked the idea of an app designed to help them decide whether, when, and how to stop smoking. They also liked the overall planned intervention content and the personal experiments feature (a key component of the experimental intervention) based on the concept for each, the component feature names, and brief descriptions of each feature; the ability to earn badge rewards; and the access to free quitline counseling and NRT through the app. One participant, however, said they would not call the free quitline because they do not like talking to counselors.

When asked which of the 10 personal experiments sounded most interesting based on their names and a brief teaser description of each, the 2 most popular (endorsed by at least half of the participants) were Track Your Triggers (5/8, 63%) and Master an Unlit Cig (4/8, 50%). There was less consensus when asked which experiments did not look interesting; none of the experiments were selected by at least half of participants. Notably, no participant identified the final practice quit attempt (Put It Into Practice) as not being of interest. Because no specific experiments were objectionable to most participants, each was maintained in the final app with minor adjustments to the teaser descriptions based on participants’ feedback.

**Figure 1:**
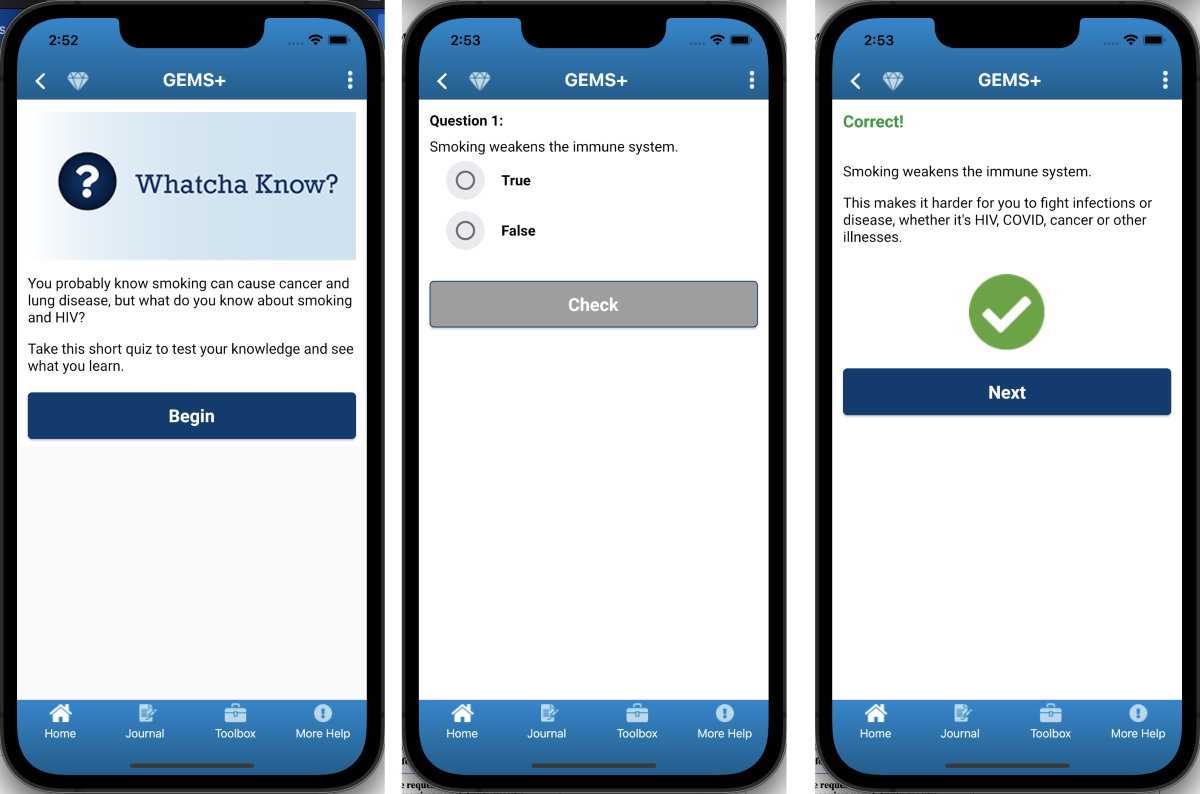
Sample screens from GEMS+ personal experiment, Whatcha Know? Content designed for people living with HIV who smoke cigarettes.

After viewing content planned for the Whatcha Know? experiment, which was structured as a short true-false quiz testing people’s knowledge of the risks of smoking and benefits of quitting when living with HIV (Figure 1), everyone (8/8, 100%) found the content informative and generally had a positive reaction (eg, “You know, when you look at something like this...seeing it written out...it has an impact whether you want it to or not.” [participant 45]). This included those who initially were not interested based on the name and brief description (2/8, 25%; “I imagine it’s all horrifying stuff I already know.” [participant 45] and “We all know why smoking is bad for us, especially with HIV.” [participant 44]). The more positive reaction after people viewed the full content reinforced inclusion of this personal experiment in the final app.

We also included information about the risks of smoking when living with HIV and benefits of quitting in other sections of the app. After viewing the planned information, 37% (3/8) of the participants questioned its veracity and several initially characterized it as “fear statements,” “scary,” and “stark.” However, after reflecting on this information, 6 (86%) of the 7 participants who viewed it said it either made them think differently about quitting smoking or that they thought it would make others think twice about smoking. Several participants also had strong reactions to the suggestion that smoking could hasten the development of AIDS and questioned the accuracy of this information. As a result, in the final app version, we included this HIV-specific risk and benefit information in a feature called, Just the Facts. Information about the impact on smoking progression was also included in a peer testimonial section using a physician peer as the information source, and the content was revised to make it clear that AIDS is not inevitable and smoking cessation can reduce risk of HIV disease progression.

Finally, we assessed participants’ reactions to 3 potential names and logos for the new app. Each name was chosen to reflect its relevance for people who are HIV positive, but without making this obvious, for confidentiality reasons: MAPPS (My Advice Program for Positive Smokers), MyPAQ (My Positive Advice about Quitting), and GEMS+. The latter is not an acronym. Instead, it was chosen to reflect that the app content was grounded in the original GEMS content with additional content tailored to people who were HIV positive, hence GEMS+. It also reflects that users can earn “gemstone” reward badges for using the app. Phase 1 participants appreciated the confidentiality of each of the names and that these names did not indicate that the app was obviously for people living with HIV. Of the 3 names, GEMS+ received the most support among the 7 participants who provided feedback and was ultimately chosen.

### Phase 2: Randomized Pilot Study

#### Participants

Demographic characteristics of the final analytic sample (n=19) are presented in Table 1, along with other relevant baseline descriptors. Participants were aged 49.3 (SD 11.6) years on average and were predominantly male (17/19, 89%) and White (13/19, 68%). Of the 19 participants, 16 (84%) identified as gay, lesbian, bisexual, or pansexual; 2 (11%) identified as heterosexual; and 1 (5%) as other. Approximately half (9/19, 47%) of the participants had a household income <US $45,000 per year, and most participants (11/19, 58%) did not have a college degree. Participants smoked half a pack of cigarettes per day on average.

**Table 1:** Phase 2 randomized pilot participants’ baseline descriptors.

Characteristics			Overall (N=19)		Control (n=7)		Experimental (n=12)
Male, n (%)			17 (90)		7 (100)		10 (83)
White, n (%)			13 (68)		6 (86)		7 (58)
Hispanic, n (%)			0 (0)		0 (0)		0 (0)
Employed^a^, n (%)			12 (63)		4 (57)		8 (67)
Annual household income <US $45,000^a^, n (%)			9 (47)		4 (57)		5 (42)
No college degree, n (%)			11 (58)		4 (57)		7 (58)
Mental health or substance use disorder^a,b^ (yes), n (%)			5 (26)		3 (43)		2 (17)
Age (y), mean (SD)			49.3 (11.6)		48.9 (10.7)		49.6 (12.5)
**Nicotine and tobacco**							
	Cigarettes per day, mean (SD)	11.3 (4.6)		10.3 (3.5)		11.9 (5.2)	
	FTND^c^ (nicotine dependence)^d^, mean (SD)	4.8 (1.4)		4.4 (1.5)		5.1 (1.3)	
	Use tobacco other than cigarettes (yes), n (%)	4 (21)		2 (29)		2 (17)	
	Use e-cigarettes (no), n (%)	2 (11)		1 (14)		1 (8)	
	Nicotine dependence: high or very high^d^, n (%)	6 (32)		2 (29)		4 (33)	
**Substance and alcohol use, n (%)**							
	Cannabis use: ≥1 times/week	3 (16)		2 (29)		1 (8)	
	Hazardous drinking levels^e^	6 (32)		3 (43)		3 (25)	
**App use history, n (%)**							
	Currently using health app (yes)	9 (47)		3 (43)		6 (50)	
	Ever downloaded smoking app^a^ (yes)	3 (16)		1 (14)		2 (17)	
**Motivation^f^, mean (SD)**							
	Reducing smoking	7.2 (2.1)		7.4 (2.5)		7.1 (2)	
	Quitting smoking	8.6 (1.4)		9 (1.4)		8.3 (1.4)	
**Self-efficacy^f^, mean (SD)**							
	Reducing smoking	6.3 (1.8)		7.1 (2)		5.8 (1.6)	
	Quitting smoking	7.2 (1.7)		8.1 (1.1)		6.7 (1.8)	

^a^Missing responses: employed (n=1), annual household income (n=3), mental health or substance use disorder (n=1), and ever downloaded smoking app (n=1).

^b^Self-reported diagnosis or treatment for depression, anxiety, bipolar disorder, schizophrenia, or alcohol use.

^c^FTND: Fagerström Test of Nicotine Dependence.

^d^FTND score: range is from 0 to 10. Scores 6 or 7 indicate high dependence and 8 or 10 indicate very high dependence.

^e^Score of ≥4 for men and ≥3 for women (out of 12) on the Alcohol Use Disorders Identification Test-Consumption score, indicating drinking levels that are hazardous to one’s health and safety.

^f^5-point Likert scale ranging from 1=“not at all” to 10=“extremely.”

#### Indices of Feasibility: Recruitment, App Installation, and Retention

Recruitment proved to be a challenge. Overall, 23 people consented and were randomized to the phase 2 pilot study. Of the 23 people, 9 (39%) were recruited from KP (out of 85 mailed invitations), 9 (39%) from ResearchMatch (out of 534 announcements and 52 requested emails with additional study information), 1 (4%) from an advertisement in a local internet-based newspaper (out of >6000 advertisement views and 273 advertisement clicks), and 3 (13%) from flyers in local community health clinics. Due to newly imposed restrictions on Facebook advertising at the time, advertisements were not able to be targeted to people living with HIV, making it harder to reach the target audience with the study’s limited advertising budget. Consequently, only 1 person was recruited from Facebook (out of 15,700 advertisement views and 1100 advertisement clicks). This person was later deemed ineligible per our fraud detection protocol and was withdrawn from the study, leaving a total of 22 eligible people. No one was successfully recruited from advertisements placed on Twitter, Grindr (Grindr Inc), or Poz [54], despite broad visibility based on tracked advertisement views for each.

**Figure 2:**
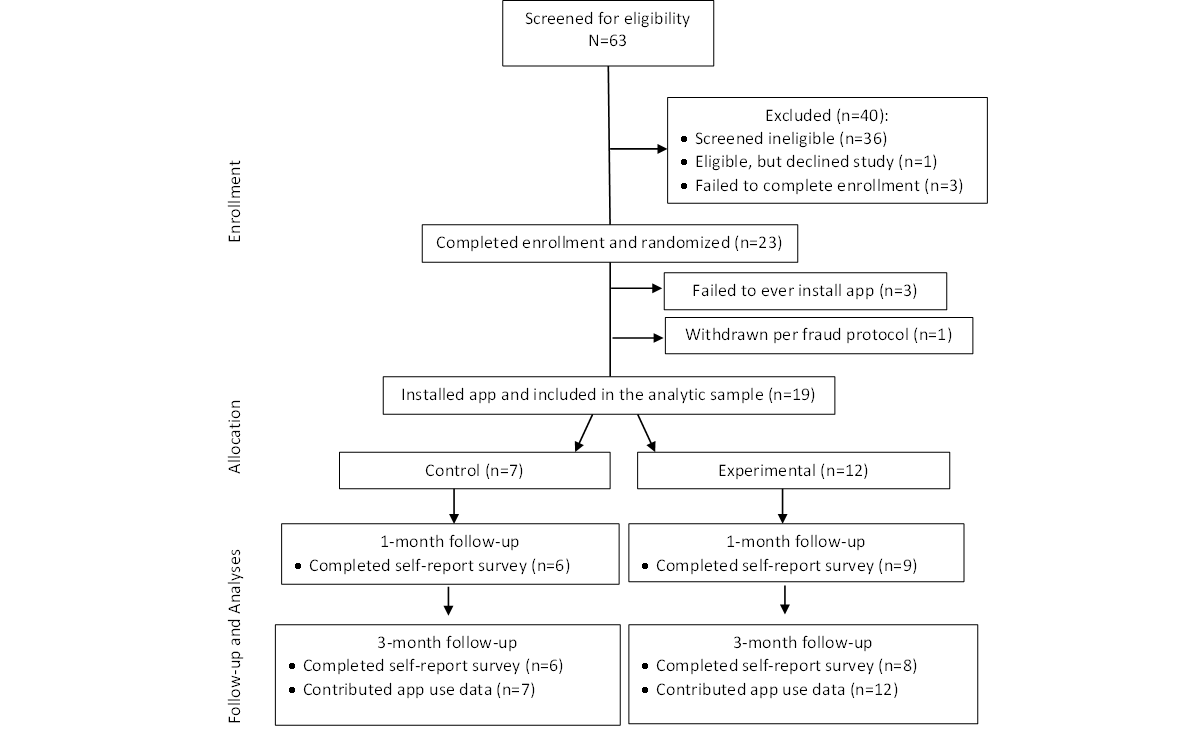
CONSORT (Consolidated Standards of Reporting Trials) flow diagram for GEMS+ randomized pilot study.

Of the consented individuals eligible to participate, 86% (19/22) installed the app and were included in the final analytic sample (Figure 2). While the target sample of 50 was not reached, recruitment was discontinued after 7 months due to budget and time constraints.

Once enrolled, participant retention was good: 74% (14/19) of the participants completed the 3-month follow-up. This included 86% (6/7) of the control participants and 67% (8/12) of the experimental participants. Retention was highest among people recruited from KP: 86% (6/7) KP members versus 67% (8/12) of the participants recruited from all other sources completed the 3-month survey.

#### Indices of Acceptability: Satisfaction and Use

Most participants—75% (3/4) of controls and 87% (7/8) of experimental participants—said they would recommend their assigned app to a friend (RD 0.1, 95% CI –0.4 to 0.7). Among those who earned at least 1 utilization badge, self-reported app satisfaction was modest in both arms but descriptively favored in the experimental arm: mean 3.6 out of 5 (SD 0.8) in the experimental arm versus 3.2 (SD 1.3) in the control arm (adjusted mean difference 0.2, 95% CI –1.3 to 1.7).

Indices of general app use favored the experimental arm. Experimental arm participants descriptively averaged more total sessions (mean 8.2, SD 6.9 vs mean 4.9, SD 1.3 for controls; adjusted mean difference 3.6, 95% CI –2.3 to 9.4) and earned more badges (mean 5.5, SD 3.9 vs mean 1.8, SD 2.1 for controls; adjusted mean difference 4.0, 95% CI 0.78-7.183), and more experimental participants earned the requisite 6 badges required to request a free trial of NRT (7/12, 58% experimental participants compared to 0/7, 0% of controls; RD 0.6, 95% CI 0.2-0.8). However, total duration of app use favored the control arm: mean 54.3 (SD 39.8) days among controls versus 30.4 (SD 20.6) days in the experimental arm (adjusted mean difference –22.1, 95% CI –53.4 to 9.2). The average among controls was influenced by 3 participants with >80 days between their initial and final day of app use. Overall use of the app features that were common in the control and experimental app versions was low (Table 2).

In the experimental arm, 25% (3/12) of the participants viewed the *Just the Facts* section, which contained information about the risks of smoking with HIV. Completion rates across the 10 personal experiments ranged from 83% (10/12) for the first personal experiment to 17% (2/12) for the last personal experiment at the 3-month follow-up (Table 3). Median ratings of helpfulness for each experiment were 3 on a 5-point Likert scale.

**Table 2:** Use of features common to both GEMS+ app versions (control and experimental).

App feature		Control (n=7), n (%)	Experimental (n=11)^a^, n (%)	Risk difference (95% CI)^b^
**Quit Guide**				
	Viewed ≥1 step	6 (86)	1 (9)	–0.766 (–0.962 to –0.303)
	Completed ≥1 step^c^	4 (57)	1 (9)	–0.481 (–0.830 to 0.002)
Cigarette tracker^d^		1 (14)	3 (25)	0.130 (–0.332 to 0.491)
Savings calculator^e^		2 (29)	3 (27)	–0.013 (–0.485 to 0.430)
Peer testimonials^f^		1 (14)	1 (9)	–0.052 (–0.471 to 0.283)
Peer advice^g^		1 (14)	2 (18)	0.039 (–0.392 to 0.390)
Journal^h^		6 (86)	8 (73)	–0.130 (–0.491 to 0.339)
More Help^i^		4 (57)	6 (55)	–0.026 (–0.476 to 0.446)
Call Now^j^		0 (0)	1 (8)	0.083 (–0.297 to 0.388)

^a^For all items except cigarette tracker, data were not available for 1 experimental arm participant due to use of virtual private network (refer to the Pilot Study Data Issues section). Total experimental sample for cigarette tracker, n=12.

^b^Risk differences calculated using arithmetic difference in sample proportions (experimental–control) accompanied by exact 95% CIs.

^c^On the basis of completion of Quit Guide step content as defined by user marking all content in section as read.

^d^On the basis of use of tracker to log smoking on at least 1 day.

^e^On the basis of use of savings calculator to estimate cost savings of quitting smoking.

^f^On the basis of event data showing each peer testimonial modeling how to talk back to common excuses for not quitting was opened and viewed.

^g^On the basis of event data showing each vignette providing motivational support and advice was opened and viewed.

^h^On the basis of event data showing the journal was opened at least one time, whether or not an entry was created.

^i^On the basis of opening the More Help page at least 1 time to access tobacco quitline referral and other information on where to get help quitting smoking.

^j^On the basis of clicking “call now” to automatically dial tobacco quitline service provider.

**Table 3:** Portion of GEMS+ experimental arm participants completing each personal experiment exercise (n=12).

Experiment	By 1 month, n (%)	By 3 months, n (%)
Your 75th Birthday	10 (83)	10 (83)
Whatcha Know?	7 (58)	9 (75)
Explore Your Motivation	5 (42)	5 (42)
Track Your Triggers	5 (42)	5 (42)
Box Breathing	4 (33)	4 (33)
Mastering the Unlit Cig	3 (25)	3 (25)
Make Smoking Boring	2 (17)	3 (25)
It’s Your Choice	2 (17)	2 (17)
Cut it Out	2 (17)	3 (25)
Put it Into Practice	1 (8)	2 (17)

#### Indices of Impact: Change in Smoking Behavior and Treatment Uptake

Indices of impact on smoking behavior also descriptively favored the experimental arm at the 3-month follow-up: 67% (8/12) of the experimental participants and 57% (4/7) of the controls reported making a quit attempt after joining the study (RD 0.1, 95% CI –0.4 to 0.6), 37% (3/8) of experimental participants compared to 20% (1/5) of the controls reported a ≥50% reduction in their baseline daily smoking (RD 0.2, 95% CI –0.4 to 0.6), and 8% (1/12) of the experimental participants versus 0% (0/7) of the controls clicked on the Call Now button to connect with a free tobacco quitline counselor (RD 0.083, 95% CI –0.297 to 0.388). However, none of the participants in either arm who earned the ability to request the free NRT did so, and no participants in either arm reported 7-day point prevalence abstinence at the 3-month follow-up.

With regard to the impact on cognitive intermediaries of behavior change, mean self-reported motivation to quit at 1-month follow-up was 6.8 (SD 2.7) for experimental participants compared to 7.5 (SD 2.5) for controls (adjusted mean difference –0.1, 95% CI –2.7 to 2.4). Motivation to smoke less at 1-month follow-up averaged 7.3 (SD 2.1) for experimental participants compared to 7.2 (SD 1.3) among controls (adjusted mean difference –0.2, 95% CI –1.6 to 1.3). Mean self-efficacy for quitting smoking was 6.2 (SD 1.5) for experimental participants compared to 8.3 (SD 2.1) among controls (adjusted mean difference –1.3, 95% CI –3.6 to 0.9). Self-efficacy for smoking less at 1-month follow-up averaged 6.3 (SD 1.1) for experimental participants compared to 5.8 (SD 1.9) among controls (adjusted mean difference 0.6, 95% CI –1.1 to 2.5).

## Discussion

### Principal Findings

We conducted a 2-phased, formative research study to inform the design and acceptability of a novel mobile health app for PAQS living with HIV and the feasibility of a future randomized efficacy trial. Due to the small samples involved in this work, all results should be considered suggestive and not definitive.

In general, the results from this work suggest PAQS living with HIV may be receptive to an app-based intervention designed to help them decide whether, when, and how to stop smoking. All participants interviewed in phase 1 expressed interest in the app concept, and all but 3 phase 2 pilot participants who enrolled in the study subsequently installed and used their assigned app. Among the 2 defined patient populations of known smokers living with HIV (KP Washington membership and ResearchMatch registry), 3.1% (18/586) of those contacted ultimately enrolled. Moreover, the proportion of participants who said they would recommend the app to a friend after using it was descriptively higher for the experimental arm than for the control arm (7/8, 87% vs 3/4, 75%). Other descriptive indices of acceptability also favored the experimental arm, including participants’ satisfaction ratings, total number of sessions completed, total number of utilization badges earned, and the proportion who earned enough badges to request a free trial of NRT. However, overall use of the app in both arms was low compared to that observed in our prior trial testing a similar experimental app among PAQS who did not have HIV [31]. In that pilot study, experimental users averaged 19.9 sessions versus 8.2 in this study and control participants averaged 7.3 sessions versus 4.9 in this study, despite using an identical control app. Given the small sample size in both studies, these proportions may not generalize to a larger population, but they could suggest that PAQS living with HIV, in general, are less interested in using an app-based smoking intervention than people who are not living with HIV. More research on this point is warranted.

Similarly, indices of the apps’ potential impact favored the experimental arm, with a descriptively higher proportion of people using the experimental app making a quit attempt, reducing their smoking, and calling the free tobacco quitline. However, no one in either arm quit smoking, unlike in our prior trial where 17.9% of the experimental participants and 3.4% of the controls called the tobacco quitline and 14.7% of the experimental participants and 6.9% of the controls quit smoking at the 3-month follow-up [31].

The study results also highlight the challenges of recruiting PAQS living with HIV. Despite our success using social media and internet-based advertising to recruit for other studies targeting PAQS [31,34,41], this was more difficult than expected in this trial. A key driver of this was the newly implemented policy limiting placing advertisements on Facebook or Instagram that targeted people with specific health conditions, such as HIV. It is also possible that recruitment was more difficult due to pandemic fatigue in 2022, which the World Health Organization described as decreased interest in engaging in protective behaviors [55]. The latter was particularly observed in response to COVID-19–related protective behaviors, but likely impacted people’s willingness to engage in a range of health behaviors, including smoking cessation or willingness to volunteer for research. Whatever the driver, this experience was not unique to our study. Most National Cancer Institute–funded researchers recruiting people living with HIV to participate in smoking cessation studies reported significant recruitment challenges during the initial years of the COVID-19 pandemic [56].

With a large enough internet advertising budget, one might be able to overcome the inherent barriers of recruiting a niche population via social media, but our experience suggests it would be more efficient to recruit from defined patient populations, such as through a health care system or patient research registry.

### Limitations and Strengths

The findings from this work must be viewed with caution due to the small sample size and formative nature of this work. The study was not powered to detect clinically meaningful differences with statistical significance, which limits our ability to draw any firm conclusions about the generalizability of the findings.

To our knowledge, GEMS+ is the first app to have been designed specifically for PAQS living with HIV. The study, thus, addresses an important intervention gap. It also addresses the “ethical imperative” of including diverse populations in digital health research [57]. Other strengths include a rigorous methodological design, which allows the unique effects of the content designed for PAQS living with HIV to be explored independently of the core control content and our ability to contrast recruitment success via the different outreach strategies tested.

### Conclusions

PAQS who are living with HIV represent an important population for smoking cessation interventions. This formative work suggests that these individuals may be receptive to using a mobile health–based app intervention to help them decide whether, when, or how to stop using tobacco. Indices of feasibility, acceptability, and impact suggest that additional research and development are warranted.

## Appendix

Multimedia Appendix 1 CONSORT eHEALTH checklist (V1.6.1).

## Abbreviations

ART: antiretroviral therapy
KP: Kaiser Permanente
MAPPS: My Advice Program for Positive Smokers
MyPAQ: My Positive Advice about Quitting
NRT: nicotine replacement therapy
PAQS: people ambivalent about quitting smoking
RD: risk difference
REDCap: Research Electronic Data Capture

## Data Availability

The data sets generated during and analyzed during this study are not publicly available because this study was not subject to the National Institutes of Health’s data sharing requirements and we do not have institutional review board approval to publicly share data, but are available from the corresponding author on reasonable request.
